# Phenolic Composition Stability and Antioxidant Activity of Sour Cherry Liqueurs

**DOI:** 10.3390/molecules23092156

**Published:** 2018-08-27

**Authors:** Anna Sokół-Łętowska, Alicja Z. Kucharska, Antoni Szumny, Katarzyna Wińska, Agnieszka Nawirska-Olszańska

**Affiliations:** 1 Department of Fruit, Vegetable and Plant Nutraceutical Technology, Wrocław University of Environmental and Life Sciences, J. Chełmońskiego 37, 51-630 Wrocław, Poland; alicja.kucharska@upwr.edu.pl (A.Z.K.); agnieszka.nawirska-olszanska@upwr.edu.pl (A.N.-O.); 2 Department of Chemistry, Wrocław University of Environmental and Life Sciences, J. Chełmońskiego 37, 51-630 Wrocław, Poland; antoni.szumny@upwr.edu.pl (A.S.); katarzyna.winska@upwr.edu.pl (K.W.)

**Keywords:** liqueurs, sour cherry, phenolic compounds, anthocyanins, aromatic compounds, antioxidant activity

## Abstract

The aim of the study was to evaluate changes of phenolic and anthocyanin contents, antioxidant activity, aroma compounds and color of sour cherry liqueurs with and without sugar during 6 months of storage at temperatures of 15 °C and 30 °C. Contents of phenolic compounds (HPLC, UPLC-MS) and antiradical activity (ABTS) changes were measured. Color changes were measured by an objective method (ColorQuest XE). During storage fluctuations of phenolic compounds and antioxidant activity content were observed. The content of substances which react with Folin-Ciocalteu reagent was comparable before and after 24 weeks. During the 24 weeks of storage, the highest average antioxidant activity against ABTS radicals was shown by sour cherry liqueurs without sugar, stored at 15 °C. Quicker degradation of anthocyanins was observed in liqueurs without sugar, stored at 30 °C (t_1/2_—5.9 weeks in liqueurs with sugar and 6.6 weeks in liqueurs without sugar). Better stability of red color was observed in liqueurs with sugar, stored at 15 °C. The content of the dominant aroma compound, benzaldehyde, increased during storage. Long-term storage and sugar addition decreases color attributes but increases organoleptic value without of great influence on antioxidant activity. Studies on a half-year period of liqueur storage showed that their characteristic features are almost unchanged if stored at 15 °C and without sugar added, but organoleptic properties were better in samples stored at 30 °C.

## 1. Introduction

Fruit liqueurs are traditional alcoholic beverages made by maceration of fruits in an aqueous solution of ethanol. The way they are prepared is important to achieve the proper flavor and aroma of liqueurs. Strength and the proportions of alcohol to the components, the method of preparation as well as time of maceration may vary (from several days to several months) [1,2,3,4]. 

Liqueurs and tinctures have been used in folk medicine for many years. Today, it is known that the therapeutic effects of liqueurs are associated with the presence of phenolic compounds, which are valuable components of the diet. Recent dietary recommendations permit small amounts of alcoholic beverages. According to the recommendations of the Dietary Guidelines for Americans for 2010–2015 and 2015–2020 [5,6], moderate consumption of alcoholic beverages may have beneficial effects on human health. Phenolic compounds are dominant compounds antioxidants in alcoholic beverages made from fruit. Their biological activity has been repeatedly confirmed. As a component of liqueurs, they are able (to some extent) to counter the pro-oxidant activity of alcohol [7]. 

Cherry fruit is characterized by a red color, juicy flesh, a sour taste and a pleasant aroma. Therefore, in many countries sour cherry liqueurs are especially popular. Cherries contain more than 200 mg of polyphenols/100 g of fruit. They are rich in flavonoids represented by anthocyanins (mainly glycosides of cyanidin), flavan-3-ols (catechin, epicatechin), flavonols (rutin, quercetin, kaempferol) and phenolic acids: chlorogenic, *p*-coumaric, caffeic. They are one of the few fruits to contain melatonin [8,9,10,11].

Cherry stones contain cyanogenic glycosides, including amygdalin. These compounds are characterized by potential toxicity after hydrolysis into hydrogen cyanide. During the process of maceration of fruit, they can be extracted into the liqueur. The concentration of these toxic ingredients in the finished product depends on the way and time of fruit maceration, but also on the time the liqueurs are stored. 

Due to the amygdalin content, especially in stones, sour cherry liqueurs should be prepared from partially pitted cherries or in a short period of extraction time to avoid the presence of excessive amount of cyanogenic glycosides in the liqueur. According to Sokół-Łętowska [12] the content of HCN decreased 5–10-fold during prolonged storage. Leaving a small amount of kernels in the solution leads to release of hydrocyanic acid from stones and imparts a gentle bitter almond flavor to the beverage.

There are very few research papers on the characteristics of fruit liqueurs. Nikicevic et al. investigated the flavors of cherry spirits made from five Serbian varieties of cherries after fruit fermentation. Ethyl octanoate and ethyl decanoate were determined in the highest concentrations in distillates [13]. Rodtjer et al. [14] investigated phenolic compounds in an aromatic bitter and a cherry liqueur by high-performance liquid chromatography (HPLC) with electrochemical detection. There is a general lack of research on phenolic compound content changes during storage. In our previous study [15] we compared ten liqueurs made from red fruit and changes in contents of the main phenolic groups during storage.

In the production of fruit liqueurs, different technologies are used (e.g., using the different order of adding ingredients, running the process at different temperatures, etc.). It is important to maintain the high quality of the product. These issues have not been tested so far, therefore it was decided to determine whether the addition of sugar during the production of liqueurs and storage temperature will affect the final quality of the product. The purpose of the work was to determine whether and how the sugar added to the fruit extract influences the quality of the obtained liqueur. 

The second aim of this study was to identify phenolic compounds and quantify the main anthocyanins, flavonoids, and phenolic acids that were found in sour cherry liqueurs stored for six months at 15 and 30 °C.

## 2. Results

### 2.1. Identification of Phenolic Compounds

Compounds identified before and after storage of cherry liqueurs are presented in Tables S1 and S2, and Figure S1. However, although LC-TOF-MS/MS was used for identification, the information provided by UV absorptions, retention times, mass and MS/MS spectra were not sufficient to determine the identity of all compounds. Identified compounds are in agreement with earlier research [16,17,18]. During storage some of the compounds disappeared or underwent rearrangements. In samples with added sugar partial disappearance of caffeoylquinic acid derivatives, protocatechuic acid hexoside and some of the dimeric and trimeric flavanols occurred. At 30 °C the isomer of coumaroylquinic acid appeared, but flavanol dimers and monomers disappeared, probably due to rearrangement of the moieties to afford polymeric forms.

#### 2.1.1. Phenolic Acids

Directly after preparation of liqueurs 188.4–203.4 µg/mL of phenolic acids were determined (Table 1). The main phenolic acids in sour cherry liqueurs were *p*-coumaroylquinic (about 53.3%), neochlorogenic (25.1%) and chlorogenic (21.6%) acids. Hydroxycinnamic acids were quite stable during storage. After 6 months of storage a concentration of 165.1–177.0 µg/mL was determined. In samples without and with sugar added the phenolic acid content was 176.0–177.0 µg/mL and 165.0–170.7 µg/mL, respectively, that is about 4–7% and 15–17% less than the initial content, respectively. Losses of coumaroylquinic acid—the main phenolic acid in cherry liqueur—were slightly greater than those of other acids (7.4–20.7%). Similar results were obtained in a previous study by Kallithraka et al. on changes in phenolic composition of white wines during storage [19].

#### 2.1.2. Flavanols

(−)-Epicatechin, procyanidins B1, B2, C1 and other unidentified dimers, trimers and tetramers were present in liqueurs (Table 2). The concentration of the main flavan 3-ol—procyanidin B2—was 80.5–90.9 µg/mL. Flavanols were unstable during storage and their content decreased significantly during 24 weeks. After storage at 15 °C the content of procyanidin B2 decreased to 53.2 µg/mL and 39.6 µg/mL. At 30 °C complete degradation was observed after 18 weeks (the sample with sugar). 

In samples stored at 30 °C the content of flavanols increased or decreased in the first 6–12 weeks and then consequently decreased due to the disappearance of monomers and dimers, faster in liqueurs with added sugar. The content of trimers and tetramers decreased, but slower than that of the monomers and dimers, probably due to polymerization of monomers and dimers, because near to 6–12 weeks of storage the content of trimers and tetramers increased and later decreased to 25–30% of the initial values. The total flavanol content in stored liqueurs was 4.7–5.9% of the initial value (at 30 °C) and 78.5% and 42.3% of initial values in samples stored at 15 °C without and with sugar respectively. Fluctuations of flavanol contents were observed during storage, as is seen for example for the sum of epicatechin and an unknown dimer (Table 2) and the sum of procyanidin C1 and a tetramer. This may be the result of rearrangement and/or hydrolysis of dimer moieties. 

#### 2.1.3. Flavonols

An amount of 139.7 to 158.6 µg/mL of flavonols was determined in liqueurs before storage and from 105.0 to 130.0 µg/mL after 24 weeks of storage (Table 3). Flavonols were more stable during storage than other groups of phenolic compounds. Our results are in agreement with data published by Nowicka and Wojdyło [16]. Differences in behavior of samples stored at 15 °C and 30 °C and with/without sugar added were observed. In liqueurs prepared without sugar more flavonols remained after storage—71.8% and 93.2% of initial content (at 30 °C and 15 °C respectively)—whilst in samples with sugar added 68.3% and 82.0% of the initial sum of flavonols was determined. The derivatives of kaempferol, quercetin and isorhamnetin were identified. The main flavonol was quercetin 3-rutinoside (48.2–52.2 µg/mL), and that compound was quite stable during storage, because after storage liqueurs contained 43.6–46.6 µg/mL. A similar observation was made for other flavonols. The content of quercetin increased after 24 weeks from 0.9 µg/mL to 2.3–4.0 µg/mL, increasing most in samples with sugar stored at 30 °C. It may be the result of hydrolysis of quercetin derivatives, as sour cherry liqueurs have high acidity, which favors such reactions.

#### 2.1.4. Anthocyanins

The content of anthocyanins immediately after preparation of sour cherry liqueurs was 216.8–230.8 µg/mL (Table 4). As expected, the amount of anthocyanins decreased significantly during storage, to 39.1–39.8% of the initial value in samples stored at 15 °C and to 6.5–6.9% in samples stored at 30 °C. The content of individual anthocyanins decreased to 58.4–33.1% at 15 °C and to 2.0%–7.9% of the initial value at 30 °C.

The proportions of the main anthocyanins—cyanidin 3-*O*-glucoside-rutinoside and cyanidin 3-*O*-rutinoside—were 62.2% and 25.6% of total anthocyanin content respectively before storage and 66.0% and 21.4% after storage at 30 °C. It means that cyanidin 3-glucoside-rutinoside (three sugar moieties) was more stable than cyanidin 3-rutinoside (two sugars). It is in agreement with literature data, indicating that the sugar moiety stabilizes anthocyanin stability [11,20,21,22].

During the first 12 weeks of storage the anthocyanin content was higher in samples with sugar added, but later the content of anthocyanins decreased faster in these liqueurs, and at the end of the experiment a slightly lower content of them was determined. This can be explained by the fact that presence of anthocyanin degradation products results in more rapid degradation [23,24]. The calculated half-life of total anthocyanins and the parameters of degradation kinetics are presented in Table 5. 

Samples without added sugar degraded a little slower than samples with added sugar, the half time being about 12% shorter in samples with sugar added. More important was the temperature of storage. At 30 °C anthocyanins degraded about 2.5 times faster than at 15 °C. The half-lives (t½) of the anthocyanins were in agreement with the Hellstrom et al. [23] research on anthocyanins in juices stored at different temperatures and the study by Bonerz et al. [11] on tart cherry juices. 

### 2.2. Color Changes

**Figure 1:**
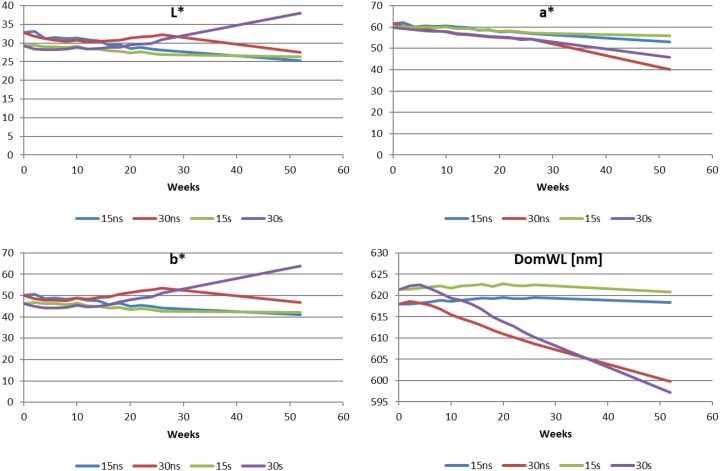
CIELAB chromatic coordinates L* (lightness), a* (red to green), b* (yellow to blue) and DomWL (dominant wavelength) and their changes during storage of liqueurs.

Cherry liqueurs were characterized by a nice red, stable color, which was due to the quite good stability of anthocyanin pigments, especially at 15 °C. The differences among samples after 26 weeks of storage were very small (Figure 1), so we decided to measure the color of liqueurs additionally after one year of storage. Color differences were visually noticeable after the annual storage.

After 24 weeks of storage, the differences in lightness ΔL* were in the range −4.4 to +0.4 units, and after one year of storage the ΔL* was between −7.6 and +8.7, and only the liqueur with sugar, stored at 30 °C, was found to brighten. The red parameter (a*) decreased after 24 weeks by 2.2–4.2 units at 15 °C and 5.8–7.1 units at 30 °C, and after 52 weeks by 4.0–8.5 and 14.2–21.6 units respectively. Larger changes in this parameter were observed for liqueurs without sugar added. The color parameter b* (yellowness) increased during storage of liqueurs at 30 °C (Δa = 2.6 ÷ 3.0 units), and decreased at 15 °C (Δa = 2.8 ÷ 5.3 units). After one year only in the sample with added sugar stored at 30 °C was an increase of parameter b* observed (Δb* = 17.4). Also, the dominant wavelength decreased, but at 15 °C values were irrelevant (ΔDomWL < 1), and at 30 °C the difference compared to initial values was approximately 10 units in the samples stored at 15 °C and 18–24 units in the samples stored at 30 °C. 

An important parameter is ΔE, describing the ability of the human eye to discriminate between the colors of products. Pérez-Magariño and González-Sanjosé [25] stated that human eyes can distinguish the color of two samples when ΔE is greater than 5 units. Liqueurs’ color changed during 24 weeks of storage from 4 (15 s) to 8.1 (15 ns) units. After one year the total color differences were from 6.4 and 14.7 for 15 s and 15 ns to 22.5 and 24.1 for 30 ns and 30 s respectively. According to Nikkhah et al. [26] the sugar concentration up to 20% stabilizes anthocyanins during storage. However, a higher concentration of sugar reduces their stability. Sokół-Łętowska et al. [4] reported that in the production of liqueurs, it is important not to exceed 30% of sugar in the solution due to unfavorable changes in color. However, temperature of storage is a key factor of the stored product.

Color changes are mainly the result of anthocyanin degradation. Therefore, the anthocyanin polymer percentage was determined in liqueurs Anthocyanin polymer content increased during storage from about 9% to 22–26% and 65–76% at 15 °C and 30 °C respectively (Table 5), increasing more in samples without sugar added.

### 2.3. Aroma Compounds

All tested cherry liqueurs had a strong and distinctive aroma. The main component was benzaldehyde, one of the 20 identified substances, representing more than 95% (Table 6). The cherry stones contain a cyanogenic glycoside, amygdalin, which when subject to enzymatic or thermal degradation releases benzaldehyde [27]. Benzaldehyde possesses a characteristic pleasant almond-like odor and is a desirable flavor attribute of cherry wine and liquors [28]. Small amounts of aromatic benzyl alcohol, benzaldehyde acetals and phenylacetaldehyde were also detected. These data are in agreement with Nikicevic [13]. Diethyl acetal and ethyl esters of benzoic, octanoic or succinic acid present in the liquor were the products of the reaction of the corresponding compounds with ethanol [29]. Small amounts of linear compounds, such as 2-octenal and 1-nonanol, were also detected. The ionones and their aromatic derivatives were also identified. Our results are in agreement with data published by Niu et al. [30] and Sun [31]. They identified a very similar profile of compounds in wine obtained from cherries. The GC-MS profile of aromatic compounds in tested cherry liqueurs indicates that sugar content, temperature and time of maceration had no major impact. The noticeable change, however, of cherry liqueurs’ scent may be associated with the formation of the corresponding diethyl acetal.

In organoleptic evaluation all sour cherry liqueur samples were characterized by the typical flavor of sour cherries. Samples stored at 15 °C were significantly less intense in smell and taste. In samples without sugar, stored at 15 °C, and liqueurs with sugar, stored at 30 °C, there was an acidic “compote” note—not desired in such products. The sample without sugar stored at 30 °C was evaluated as the worst in terms of both taste and smell. The liqueur made with sugar and stored at 15 °C had the best flavor. Unfortunately, there was a characteristic bitter almonds note only during the first weeks of storage, maybe because of the short time of alcohol-pits contact during liqueur preparation, which is in agreement with Loch et al. [32].

### 2.4. Changes in Antioxidant Activity

Antioxidant activity of liqueurs was measured by two methods: ABTS and by determination of substances which react with Folin-Ciocalteu reagent (FC) (Table 7). 

During 24 weeks of storage only small changes in antioxidant activity were observed. Total phenolic content before storage was at the level of 1.15 mg GAE/mL and 1.10–1.14 or 1.21–1.25 µg GAE/mL after storage in samples without and with sugar respectively. Our results are in agreement with other authors. Heinonen et al. [33] showed that cherry liqueurs contain about 1.08 mg of gallic acid equivalents in 1 mL, while Rødtjer et al. [14] determined 1.52 mg GAE/mL. Transformations of anthocyanins and other liqueur components during storage lead to polymerization and formation of new molecules that can react with Folin-Ciocalteu reagent.

Piljac-Žegarac et al. [34] studied fluctuations of phenolic compound content and antioxidant capacity during storage of fruit juices and found that all analyzed fruit juices exhibited substantial fluctuations in the FC content and antioxidant capacity during 29-day storage. Fluctuations in the content of polyphenolic compounds during storage of liqueurs of myrtle were also observed by Montoro et al. [35]. Kallithraka et al. [19] found an increase in the content of phenolic compounds after 9 months of storage of white wines as compared to 3 and 6 months. Klimczak et al. [36] found an increase of polyphenols in citrus juices after 6 months of storage. 

The antioxidant activity of cherry liqueurs measured by the ABTS test changed during storage. Average antioxidant activity before storage was 11.6–12.6 µM TE/1 mL (ABTS) and after storage it decreased by 6.4–9.2% in samples stored at 15 °C and by 19.4–37.8% in samples stored at 30 °C, decreasing more in liqueurs with added sugar. A similar effect was observed by Montoro et al. [35] in myrtle liqueurs, and was explained by hydrolysis of the flavonoid glycosides, which give an additional hydroxyl group which can participate in the reaction with the ABTS cation radical. Antioxidant activity fluctuations were observed by Sokół-Łętowska et al. during storage of sour cherry liqueurs made with different amounts of sugar [4]. The high antioxidant activity is in agreement with the high content of phenolic compounds in berries. Correlation coefficients (R^2^) of ABTS and phenolic compounds were 0.80 (flavan-3-ols), 0.78 (phenolic acids), 0.91 (flavonols) and 0.85 (anthocyanins).

### 2.5. Principal Component Analysis

Principal component analysis (PCA) was conducted to confirm some relationships among analyzed variables. PCA was performed using Statistica 13.1 software on mean values of 20 samples (liqueurs stored for 0, 6, 12, 18 and 24 weeks with and without sugar at 15 °C and 30 °C) and 19 variables (phenolic components, color attributes and antioxidant activity) for detecting the most important factors of variability and to describe the relationship between variables and observations. The first principal component (PC1) accounted for 63,98% of the variability in the data set. The second principal component accounted for 17.67% of the variance in the data set. The sum of 81.65% is high enough for a good presentation of multidimensional space in the two-dimensional projection. 

**Figure 2:**
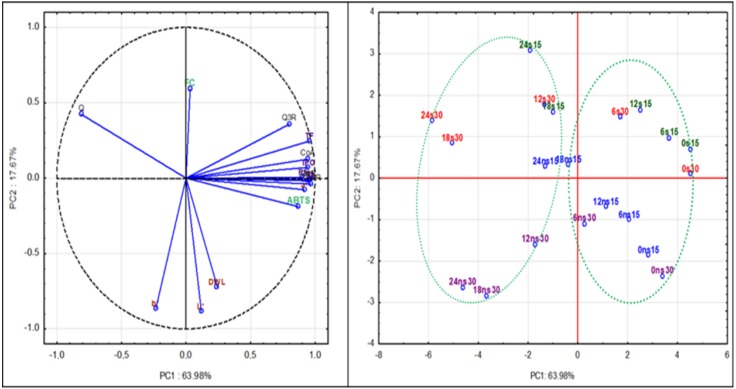
Principal Component Analysis (PCA) loadings plot and score plot of major components, color attributes and antioxidant activity of cherry liqueurs with (s) and without sugar (ns) stored at 15 °C (15) and 30 °C (30) during 24 weeks (0, 6, 12, 18, 24).

The loading plot PC2 versus PC1 (Figure 2) shows a good clustering of the objects according to the groups defined (components, color attributes and antioxidant activity) although not all groups are completely separated. On the right side of the plot are phenolic compounds. It indicating that all are very similar. Only one variable referred to quercetin lays in the left side. Quercetin appears probably as a result of the decomposition of quercetin derivatives in stored liqueurs. Antioxidant activity (ABTS) is correlated with phenolic compounds. Color Parameter a* is located near phenolic compound cluster as redness od liqueurs is strongly connected with anthocyanin content. Color attributes: b*, L* and DWL are associated with PC2 and these variables are almost not correlated with phenolic composition of liqueurs. The FC variable (substances reacting with Folin-Ciocalteu reagent) is not good parameter for estimation of polyphenol changes during storage. 

Liqueurs samples prepared with and without sugar and at different temperatures can be distinguished in the scores plot. Storage longer than 12 weeks negatively correlated with antioxidant activity and phenolic compound contents. In addition, the early, and late stages of the storage can be distinguished as indicated by the green lines. Liqueurs with sugar are well separated, by being on positive region of PC2, from liqueurs without sugar laying on negative region of PC2.

## 3. Materials and Methods

### 3.1. Preparation of Liqueurs

The sour cherries used to produce liqueurs were purchased from local market retailers and stored at −20 °C before preparation of liqueurs. The fruit (2 kg) was soaked in glass jars with the same mass of ethanol (65% vol.). Process was carried out at room temperature for three weeks without light. The contents of the jars were stirred gently every day. After 3 weeks of maceration, the fruit extract was separated from the fruit and divided into two parts. To one of them sugar was added in the amount of ¼ of the extract mass, and to the other one the same mass of water. After the dissolution of sugar each liqueur was divided into two parts and stored at temperatures of 15 °C and 30 °C for 6 months. The liqueurs prepared with sugar were labeled 15 s and 30 s, and those without sugar—15 ns and 30 ns—according to the storage temperature of 15 °C and 30 °C. The experiment was carried out in two technological duplicates. 

### 3.2. Identification of Phenolic Compounds by LC-MS Method

Identification of compounds was performed on an ultra-performance liquid chromatography (Acquity UPLC System) connected to a quadrupole time of flight (Q-TOF) MS instrument (UPLC/Synapt Q-TOF MS, Waters Corp., Milford, MA, USA) equipped with an electrospray ionization (ESI) source. Separation was achieved on the Acquity BEH C18 column (100 mm × 2.1 mm i.d., 1.7 µm; Waters, Milford, MA, USA). 4.5% formic acid in water was used as the eluent A and acetonitrile as eluent B. The gradient program was as follows: 0 min—99% (A), 12 min—75% (A), 12.5 min—100% (B), 13.5 min—99% (A). The flow rate was 0.45 cm^3^/min and the injection volume was 5 μL. The column temperature was 30 °C. The major the Q-TOF MS operating parameters were: capillary voltage, 3.0 kV; cone voltage, 40 V; cone gas flow of 11 L/h; collision energy, 50 eV; source temperature, 100 °C; desolvation temperature, 250 °C; collision gas, argon; desolvation gas (nitrogen) flow rate, 600 L/h; data acquisition range, *m*/*z* 100–1500 Da; ionization mode, negative. The data were collected by Mass-Lynx v. 4.1. software (Waters, Milford, MA, USA) [16].

### 3.3. Quantitative Analysis of Phenolic Compounds by HPLC Method

The contents of anthocyanins (TA), phenolic acids (PA), procyanidins and flavan-3-ols (TPC), flavonols (TF), and their derivatives were calculated on the basis of HPLC-PDA assays as described previously by Sokół-Łętowska et al. [15]. Phenolic compounds were monitored at 320 nm for hydroxycinnamic acids, 360 nm for flavonols, 280 nm for flavanols and 520 nm for anthocyanins. The content of anthocyanins, flavonols, flavanols, and hydroxycinnamic acids were calculated based on calibration curves determined experimentally the content of anthocyanins was converted into cyanidin 3-*O*-glucoside, flavonols into quercetin 3-rutinoside, flavanols into (+) catechin and hydroxycinnamic acids into chlorogenic acid. Analyses were performed in three repetitions. The results were expressed as µg/mL. 

The calibration curves were obtained by the external standard method on six levels of concentration of standard compounds (cyanidin 3-*O* glucoside—Extrasynthese, Lyon Nord, France, quercetin 3-*O* rutinoside, (+) catechin and chlorogenic acid—Sigma Aldrich, Steinheim, Germany), with three injections per level (Table 8). Chromatogram peak areas were plotted against the known concentrations of the standard solutions. Linear regression equations were calculated by the least squares method. As the regression coefficients R^2^ were ≥0.995, the relations were considered linear, and thus acceptable for quantifying the compounds.

RSD values of HPLC method were 0.36–7.14 for interday and intraday. Recovery values were 98.10–105.94% (Table S3). It shows, that method was reproducible and accurate.

### 3.4. Determination of Antioxidant Activity

The antioxidant activity of cherry liqueurs was determined using the Trolox equivalent antioxidant capacity (TEAC) with ABTS cation radical and was carried out according to Re et al. [37], TEAC results are expressed as µM of Trolox equivalents (TE) per 1 mL of liqueur. Content of substances which react with the Folin-Ciocalteu reagent (FC) were determined by the Folin–Ciocalteu method using gallic acid (GA) as a standard for the calibration curve. The results of the assay were calculated and expressed as milligrams of GA equivalent (GAE) per 1 mL of liqueur. The data were read at spectrophotometer (Shimadzu UV-2401 PC, Kyoto, Japan). Data are reported as the mean of three measurements.

### 3.5. Anthocyanins Polymers 

Polymerized anthocyanin determination was performed with the sodium bisulfite method according to Wrolstad [38]. The results of the assay were calculated and expressed as percent of polymeric anthocyanins.

### 3.6. GC/MS Analysis

The chemical composition of the volatile compounds was performed according to the procedure described by Tešević [39]. Briefly, liquors (80 mL) were mixed with ultrapure water (100 mL) containing sodium chloride (4 g) and then with dichloromethane (50 mL). 2-Undecanone (1 µL) was added as an internal standard for semi-quantification. Next, the mixture was stirred for 30 min using a magnetic stirrer. The layers were separated, and the organic layer was dried (2 h) over anhydrous sodium sulfate. The extract was concentrated to 1.0 mL under a gentle stream of nitrogen and kept at −27 °C in vials until the analysis. Identification and quantification of compounds present in the liqueurs were performed using gas chromatography (GC, Chrompack CP-3380 with FID detection, Varian, Walnut Creek, CA, USA) and gas chromatography/mass spectrometry (GC/MS) (Chrompack Saturn 2000, Varian, Wallnut Creek, CA, USA) with a a TRACE TR-5 column (5% phenyl methylpolysiloxane, 30 m × 0.53 mm i.d., 0.25 μm film, Thermo Fisher Scientific, Waltham, MA, USA). Scanning was performed at 1 scan s^−1^ from m/z 39 to 400 in the electron impact (EI) mode at 70 eV. helium was used as the carrier gas at a flow rate of 1 mL min^−1^ in a split ratio of 1:20 and the following temperature program: rate of 5 °C min^−1^ from 80 to 200 °C; rate of 25 °C min^−1^ from 200 to 280 °C; final hold at 280 °C for 5 min. The injector and detector were kept at 200 and 300 °C respectively. Most compounds were identified by means of three methods: (1) Kováts indices; (2) GC/MS retention times (authentic standards); (3) mass spectra with similarity indices >90% (NIST Mass Spectral Search Program Version 2.0d, Gaithersburg, MD, USA).

### 3.7. Color

The color of the liqueurs was determined with an on-camera instrumental method on the ColorQuest (HunterLab, Reston, VA, USA) apparatus, on the L*a*b* scale, D65 illuminant and observer 10°, transmitted light. Color of cherry liqueurs was additionally measured after one year of storage.

### 3.8. Organoleptic Evaluation

Organoleptic analyses were carried out by a team of five trained tasters, always the same persons to assess flavor and taste.

### 3.9. Kinetics

Degradation kinetics of main phenolic compounds during storage are obtained by determining the rate constants (k) at a given temperature against the time and half-life (t_1/2_). Degradation of phenolic compounds was considered as first-order reaction. The parameters of degradation kinetics were calculated using the following equations:

(1) $$\;k=\frac{1}{t}\ln\left(\frac{c_{0}}{c_{t}}\right)$$

(2) $$t_{1/2}=\frac{-\ln\left(0,5\right)}{k},$$

where c*_t_* is the compound concentration (µg/mL) at time t (min), c_0_ is the initial concentration (t = 0), k is the reaction rate constant.

### 3.10. Statistical Analysis

Correlation analysis and principal component analysis (PCA) was performed using Statistica (data analysis software system), version 13.1 (StatSoft, Tulsa, OK, USA) on mean values of 20 samples (liqueurs stored for 0, 6, 12, 18 and 24 weeks with and without sugar at 15 °C and 30 °C) and 19 variables: time (w0, w6, w12, w18, w24), temperature of storage (t15, t30), sugar addition (s, ns), antioxidant activity (ABTS), substances reacting with the Folin-Ciocalteu reagent (FC), procyanidin B2 (B2) and total procyanidins and flavan-3-ols (TPC), quercetin 3-rutinoside (Q3R), quercetin (Q), total flavonols (FL), coumaroylquinic acid (CoQA) and total phenolic acids (PA), cyanidin 3-*O*-glucosyl-rutinoside (C3RG), cyanidin 3-*O*-rutinoside (C3R) and total anthocyanins (AN), lightness (L*), redness (a*), yellowness (b*) and dominant wavelength (DWL).

## 4. Conclusions

This study is a contribution to knowledge about the phenolic compounds and antioxidant activity changes of sour cherry liqueurs. Their antiradical activity against ABTS is strongly correlated with the contents of flavonols, anthocyanins, flavanols and phenolic acids. Studies after a half-year liqueur storage period showed that their characteristic features are almost unchanged if stored at 15 °C and without added sugar, but organoleptic properties were better in samples stored at 30 °C. It is best to make a cherry liqueur with no added sugar and store it at 15 °C. This gives the product a good color and a high content of active compounds. Long-term (i.e., longer than half a year) storage and sugar addition reduce the color attributes but increase the organoleptic value without a great influence on antioxidant activity. Our results suggest that it might be interesting to study other technological variables of the production process, as well as different varieties of cherries.

## Figures and Tables

**Table 1:** Changes in phenolic acids content (µg/mL) during cherry liqueurs storage.

Compounds	Week	Temperature 15 °C		Temperature 30 °C	
		Without Sugar Added	With Sugar Added	Without Sugar Added	With Sugar Added
Neochlorogenic acid	0	47.61 ± 0.39 ^1^	50.85 ± 0.42	47.81 ± 0.64	50.43 ± 0.36
	6	47.66 ± 0.40	50.87 ± 0.11	46.16 ± 0.49	50.16 ± 0.47
	12	45.88 ± 0.43	49.27 ± 0.13	43.83 ± 0.59	46.47 ± 0.61
	18	46.47 ± 0.58	41.75 ± 0.00	44.33 ± 0.13	42.64 ± 0.17
	24	45.73 ± 0.16	40.81 ± 0.24	43.44 ± 0.25	42.55 ± 0.39
Coumaroylquinic acid	0	100.35 ± 0.09	108.24 ± 1.13	101.93 ± 0.65	107.52 ± 0.53
	6	100.98 ± 0.23	108.03 ± 0.02	98.93 ± 0.25	106.72 ± 0.72
	12	97.82 ± 0.83	105.16 ± 0.74	94.29 ± 1.08	99.48 ± 0.66
	18	94.62 ± 0.11	96.81 ± 1.15	89.74 ± 0.52	83.8 ± 1.19
	24	92.9 ± 1.22	92.98 ± 0.34	89.53 ± 0.00	85.25 ± 1.12
Chlorogenic acid	0	40.43 ± 0.27	44.35 ± 0.47	41.63 ± 0.57	43.41 ± 0.26
	6	40.76 ± 0.42	43.32 ± 0.28	39.95 ± 0.44	43.43 ± 0.01
	12	39.79 ± 0.47	42.98 ± 0.26	37.9 ± 0.31	40.77 ± 0.22
	18	38.63 ± 0.51	40.59 ± 0.03	44.46 ± 0.30	36.85 ± 0.16
	24	37.55 ± 0.19	36.9 ± 0.26	44.06 ± 0.04	37.27 ± 0.44
Sum of Phenolic acids	0	188.40 ± 1.15	203.43 ± 0.52	191.36 ± 1.76	201.36 ± 0.00
	6	189.41 ± 2.25	202.22 ± 0.31	185.03 ± 1.99	200.31 ± 0.11
	12	183.49 ± 2.57	197.41 ± 1.40	176.02 ± 2.14	186.73 ± 0.77
	18	179.72 ± 1.17	179.15 ± 0.35	178.53 ± 1.59	163.29 ± 1.52
	24	176.18 ± 0.42	170.69 ± 1.86	177.03 ± 1.35	165.06 ± 1.63

^1^ Values are expressed as the mean (*n* = 3) ± standard deviation.

**Table 2:** Changes in flavanols content (µg/mL) during cherry liqueurs storage.

Compounds	Week	Temperature 15 °C		Temperature 30 °C	
		Without Sugar Added	With Sugar Added	Without Sugar Added	With Sugar Added
Procyanidin B1	0	7.73 ± 0.03 ^1^	6.68 ± 0.01	5.64 ± 0.01	6.17 ± 0.05
	6	7.28 ± 0.03	5.64 ± 0.02	5.88 ± 0.06	7.21 ± 0.06
	12	4.35 ± 0.04	5.47 ± 0.01	4.99 ± 0.00	4.89 ± 0.05
	18	tr	tr	tr	nd
	24	tr	tr	0.00 ± 0.00	nd
Procyanidin B2	0	80.46 ± 0.32	90.88 ± 1.11	82.54 ± 1.13	89.84 ± 0.50
	6	75.72 ± 0.02	84.67 ± 1.17	68.89 ± 0.48	73.66 ± 0.51
	12	70.68 ± 0.08	78.14 ± 0.35	55.89 ± 0.51	58.93 ± 0.71
	18	55.34 ± 0.76	37.85 ± 0.32	31.49 ± 0.06	0.00 ± 0.00
	24	53.22 ± 0.71	39.64 ± 0.51	0.00 ± 0.00	0.00 ± 0.00
(−)-Epicatechin + dimer	0	33.03 ± 0.01	67.33 ± 0.80	50.83 ± 0.30	54.81 ± 0.75
	6	46.17 ± 0.56	50.59 ± 0.25	44.03 ± 0.57	48.73 ± 0.34
	12	37.21 ± 0.28	54.38 ± 0.57	38.11 ± 0.34	46.97 ± 0.51
	18	44.31 ± 0.41	28.04 ± 0.34	0.00 ± 0.00	0.00 ± 0.00
	24	48.17 ± 0.24	28.69 ± 0.24	0.00 ± 0.00	0.00 ± 0.00
Procyanidin C1 + tetramer	0	28.70 ± 0.09	33.21 ± 0.32	32.45 ± 0.30	31.14 ± 0.07
	6	25.72 ± 0.28	35.01 ± 0.24	32.69 ± 0.02	38.65 ± 0.40
	12	30.44 ± 0.39	34.42 ± 0.36	24.21 ± 0.33	30.66 ± 0.22
	18	13.67 ± 0.07	14.78 ± 0.05	11.07 ± 0.04	12.65 ± 0.08
	24	15.78 ± 0.05	15.39 ± 0.11	8.08 ± 0.05	10.79 ± 0.08
Sum of flavan 3-ols	0	149.92 ± 1.42	198.11 ± 1.74	171.46 ± 1.84	181.96 ± 2.11
	6	154.89 ± 0.88	175.90 ± 1.72	151.50 ± 0.17	168.24 ± 2.26
	12	142.68 ± 2.00	172.42 ± 0.73	123.20 ± 0.05	141.45 ± 0.74
	18	113.85 ± 0.61	80.84 ± 1.14	42.86 ± 0.24	12.65 ± 0.10
	24	117.67 ± 0.95	83.88 ± 0.93	8.08 ± 0.06	10.79 ± 0.10

^1^ Values are expressed as the mean (*n* = 3) ± standard deviation, tr—traces, nd—not detected.

**Table 3:** Changes in flavonols content (µg/mL) during cherry liqueurs storage.

Compounds	Week	Temperature 15 °C		Temperature 30 °C	
		Without Sugar Added	With Sugar Added	Without Sugar Added	With Sugar Added
Kaempferol-trihexoside 1	0	22.29 ± 0.14 ^1^	28.35 ± 0.18	25.63 ± 0.31	28.16 ± 0.12
	6	20.41 ± 0.28	24.74 ± 0.07	13.83 ± 0.13	16.18 ± 0.19
	12	18.29 ± 0.01	21.79 ± 0.15	8.47 ± 0.10	10.28 ± 0.09
	18	22.07 ± 0.14	18.94 ± 0.03	7.32 ± 0.06	5.34 ± 0.06
	24	18.86 ± 0.16	16.87 ± 0.17	3.93 ± 0.05	3.54 ± 0.04
Kaempferol-trihexoside 2	0	7.63 ± 0.01	11.45 ± 0.14	10.42 ± 0.10	11.29 ± 0.05
	6	6.55 ± 0.08	9.59 ± 0.12	4.39 ± 0.04	5.38 ± 0.06
	12	7.91 ± 0.07	7.83 ± 0.04	1.12 ± 0.00	2.05 ± 0.03
	18	6.27 ± 0.05	6.90 ± 0.03	1.64 ± 0.00	5.34 ± 0.01
	24	0.00 ± 0.00	5.91 ± 0.06	0.00 ± 0.00	3.54 ± 0.03
Kaempferol-dihexoside	0	16.98 ± 0.01	18.38 ± 0.01	17.24 ± 0.05	18.20 ± 0.23
	6	17.23 ± 0.01	18.53 ± 0.01	16.84 ± 0.08	18.40 ± 0.01
	12	16.64 ± 0.07	17.97 ± 0.06	16.11 ± 0.13	17.35 ± 0.09
	18	15.80 ± 0.02	15.82 ± 0.07	15.44 ± 0.05	15.12 ± 0.06
	24	15.51 ± 0.15	15.72 ± 0.19	15.34 ± 0.11	15.25 ± 0.09
Quercetin-rutinoside-rhamnoside	0	3.79 ± 0.01	3.54 ± 0.05	3.34 ± 0.01	3.51 ± 0.04
	6	3.35 ± 0.01	3.58 ± 0.03	3.27 ± 0.01	3.56 ± 0.02
	12	3.21 ± 0.01	3.44 ± 0.04	3.14 ± 0.02	3.43 ± 0.00
	18	3.32 ± 0.00	3.28 ± 0.02	3.32 ± 0.01	2.57 ± 0.03
	24	3.28 ± 0.03	3.29 ± 0.04	2.83 ± 0.03	2.53 ± 0.02
Quercetin-rutinoside	0	48.28 ± 0.20	52.15 ± 41	48.16 ± 0.27	51.71 ± 0.34
	6	48.36 ± 0.40	52.21 ± 0.52	47.58 ± 0.65	52.78 ± 0.32
	12	47.01 ± 0.08	51.06 ± 0.60	46.28 ± 0.63	49.23 ± 0.02
	18	45.64 ± 0.54	46.61 ± 0.65	43.64 ± 0.30	43.78 ± 0.03
	24	44.29 ± 0.28	46.30 ± 0.10	43.35 ± 0.59	46.69 ± 0.22
Quercetin-glucoside	0	5.90 ± 0.07	6.48 ± 0.07	5.37 ± 0.07	6.55 ± 0.07
	6	5.52 ± 0.03	6.57 ± 0.03	5.65 ± 0.07	6.71 ± 0.00
	12	6.04 ± 0.01	6.32 ± 0.01	6.17 ± 0.01	5.91 ± 0.06
	18	5.83 ± 0.05	5.91 ± 0.06	5.79 ± 0.04	5.64 ± 0.07
	24	5.68 ± 0.08	6.01 ± 0.08	6.01 ± 0.05	6.26 ± 0.02
Kaempferol-rutinoside	0	12.42 ± 0.02	14.19 ± 0.18	13.43 ± 0.00	14.25 ± 0.06
	6	13.32 ± 0.04	14.46 ± 0.11	13.26 ± 0.13	14.52 ± 0.07
	12	12.90 ± 0.06	14.17 ± 0.18	12.72 ± 0.08	13.60 ± 0.06
	18	12.39 ± 0.10	12.72 ± 0.04	11.81 ± 0.12	12.02 ± 0.11
	24	13.92 ± 0.10	12.48 ± 0.02	11.63 ± 0.12	12.68 ± 0.13
Isorhamnetine-rutinoside	0	20.92 ± 0.24	23.11 ± 0.20	21.58 ± 0.21	22.75 ± 0.03
	6	21.41 ± 0.14	23.25 ± 0.16	21.37 ± 0.14	23.33 ± 0.05
	12	20.77 ± 0.06	22.68 ± 0.15	20.45 ± 0.20	21.93 ± 0.23
	18	20.38 ± 0.09	20.76 ± 0.15	19.46 ± 0.07	19.75 ± 0.05
	24	20.11 ± 0.04	20.46 ± 0.19	19.30 ± 0.10	12.68 ± 0.16
Quercetin	0	0.88 ± 0.00	0.90 ± 0.00	0.97 ± 0.00	0.94 ± 0.00
	6	1.62 ± 0.00	1.53 ± 0.00	2.01 ± 0.02	2.43 ± 0.02
	12	1.15 ± 0.01	1.47 ± 0.00	1.76 ± 0.01	3.38 ± 0.01
	18	2.17 ± 0.02	2.06 ± 0.03	2.04 ± 0.01	2.76 ± 0.03
	24	2.30 ± 0.03	2.97 ± 0.03	2.58 ± 0.00	4.34 ± 0.05
Sum of flavonols	0	139.73 ± 0.79	158.56 ± 1.48	146.16 ± 0.54	157.38 ± 0.87
	6	138.85 ± 0.04	154.45 ± 1.62	128.21 ± 0.33	143.30 ± 1.30
	12	132.56 ± 0.73	146.72 ± 0.62	116.23 ± 0.77	127.18 ± 1.10
	18	135.50 ± 1.15	133.02 ± 0.19	110.46 ± 0.41	112.33 ± 1.38
	24	130.21 ± 1.10	130.01 ± 0.96	104.97 ± 1.05	107.52 ± 1.46

^1^ Values are expressed as the mean (*n* = 3) ± standard deviation.

**Table 4:** Changes in anthocyanins content (µg/mL) during cherry liqueurs storage.

Compounds	Week	Temperature 15 °C		Temperature 30 °C	
		Without Sugar Added	With Sugar Added	Without Sugar Added	With Sugar Added
Cyanidin 3-*O*-sophoroside	0	19.11 ± 0.13 ^1^	20.21 ± 0.10	18.98 ± 0.01	20.15 ± 0.24
	6	15.57 ± 0.07	17.17 ± 0.18	7.95 ± 0.04	9.37 ± 0.10
	12	12.32 ± 0.01	14.57 ± 0.15	3.81 ± 0.02	4.74 ± 0.01
	18	8.12 ± 0.00	12.50 ± 0.16	1.57 ± 0.02	2.81 ± 0.02
	24	6.91 ± 0.09	11.80 ± 0.06	0.88 ± 0.01	1.59 ± 0.01
Cyanidin 3-*O*-glucosyl rutinoside	0	135.23 ± 0.84	143.26 ± 1.07	134.83 ± 1.79	142.62 ± 1.03
	6	113.56 ± 0.29	125.12 ± 0.85	64.42 ± 0.91	75.92 ± 0.24
	12	92.85 ± 0.30	105.45 ± 1.31	35.04 ± 0.12	43.86 ± 0.61
	18	65.27 ± 0.61	55.17 ± 0.71	17.06 ± 0.16	15.50 ± 0.14
	24	56.14 ± 0.71	48.93 ± 0.23	11.01 ± 0.08	9.82 ± 0.01
Cyanidin 3-*O*-glucoside	0	5.18 ± 0.01	6.06 ± 0.08	5.54 ± 0.01	6.13 ± 0.01
	6	4.30 ± 0.05	4.77 ± 0.03	1.73 ± 0.01	2.30 ± 0.01
	12	3.17 ± 0.02	4.55 ± 0.01	0.68 ± 0.00	0.89 ± 0.00
	18	2.75 ± 0.02	2.44 ± 0.02	0.21 ± 0.00	0.37 ± 0.00
	24	1.76 ± 0.02	2.01 ± 0.02	0.11 ± 0.00	0.15 ± 0.00
Cyanidin 3-*O*-sambubioside-5-rhamnoside	0	2.21 ± 0.02	2.70 ± 0.00	2.54 ± 0.01	2.85 ± 0.04
	6	2.33 ± 0.02	2.31 ± 0.03	0.87 ± 0.00	1.33 ± 0.01
	12	1.60 ± 0.02	2.54 ± 0.03	0.38 ± 0.00	0.59 ± 0.01
	18	1.73 ± 0.02	1.46 ± 0.01	tr	0.31 ± 0.00
	24	1.18 ± 0.01	1.21 ± 0.01	tr	tr
Cyanidin 3-*O*-rutinoside	0	55.55 ± 0.05	58.62 ± 0.60	54.97 ± 0.13	58.58 ± 0.31
	6	44.24 ± 0.33	48.64 ± 0.43	21.75 ± 0.01	26.15 ± 0.30
	12	33.87 ± 0.29	40.34 ± 0.47	10.3 ± 0.11	13.52 ± 0.17
	18	25.38 ± 0.13	26.29 ± 0.18	4.95 ± 0.00	5.70 ± 0.03
	24	20.49 ± 0.13	22.66 ± 0.12	2.95 ± 0.02	3.19 ± 0.02
Sum of anthocyanins	0	217.28 ± 0.74	230.84 ± 2.32	216.85 ± 2.64	230.33 ± 1.50
	6	180.00 ± 1.99	198.01 ± 0.20	96.72 ± 0.23	115.07 ± 0.41
	12	143.81 ± 0.63	167.46 ± 1.66	50.20 ± 0.09	63.61 ± 0.68
	18	103.26 ± 0.38	101.42 ± 1.35	23.93 ± 0.07	24.68 ± 0.18
	24	86.48 ± 0.54	90.19 ± 0.78	15.05 ± 0.14	14.88 ± 0.07

^1^ Values are expressed as the mean (*n* = 3) ± standard deviation, tr-traces.

**Table 5:** Changes of polymeric anthocyanin percentage and the parameters of degradation kinetics of sour cherry liqueurs during storage.

	Week	Temperature 15 °C		Temperature 30 °C	
		Without Sugar Added	With Sugar Added	Without Sugar Added	With Sugar Added
Polymeric anthocyanin percentage	0	9.5	8.9	9.3	9.2
	8	12.6	10.7	20.5	18.5
	16	22.7	20.7	57.5	42
	24	25.8	21.9	75.8	65.3
k (1/week)		0.042	0.048	0.105	0.118
t_1/2_ (week)		16.4	14.5	6.6	5.9
R^2^		0.994	0.969	0.996	0.993

**Table 6:** Aroma compounds quantified in cherry liqueurs (µg/100 mL).

Compounds	tR (min)	Week	Temperature 15 °C		Temperature 30 °C	
			Without Sugar Added	With Sugar Added	Without Sugar Added	With Sugar Added
Benzaldehyde	6.85	6	9491.0 ± 54.76 ^1^	10,800.0 ± 106.30	10,150.0 ± 44.79	8600.0 ± 24.32
		24	10,900.0 ± 54.26	11,500.0 ± 19.52	12,600.0 ± 51.32	12,600.0 ± 25.66
1-Octanol	8.60	6	5.0 ± 0.04	10.0 ± 0.01	10.0 ± 0.01	30.0 ± 0.17
		24	30.0 ± 0.00	40.0 ± 0.29	20.0 ± 0.03	60.0 ± 0.68
Hexyl acetate	8.67	6	4.0 ± 0.01	40.0 ± 0.34	20.0 ± 0.04	20.0 ± 0.17
		24	20.0 ± 0.04	68.0 ± 0.29	35.0 ± 0.15	54.0 ± 0.01
Benzyl alcohol	9.64	6	45.0 ± 0.38	60.0 ± 0.25	30.0 ± 0.13	48.0 ± 0.02
		24	78.0 ± 0.43	47.0 ± 0.13	35.0 ± 0.27	47.0 ± 0.15
Limonene	10.05	6	1.0 ± 0.00	10.0 ± 0.06	1.0 ± 0.00	1.0 ± 0.00
		24	1.0 ± 0.00	1.0 ± 0.01	1.0 ± 0.00	1.0 ± 0.01
2-Octenal	10.56	6	1.0 ± 0.01	1.0 ± 0.01	1.0 ± 0.00	1.0 ± 0.01
		24	1.0 ± 0.01	1.0 ± 0.00	1.0 ± 0.01	1.0 ± 0.01
1-Nonanol	11.55	6	2.0 ± 0.00	2.0 ± 0.02	3.0 ± 0.00	3.0 ± 0.02
		24	2.0 ± 0.02	1.0 ± 0.01	3.0 ± 0.00	2.0 ± 0.02
Unknown	11.97	6	0.0 ± 0.00	1.0 ± 0.00	0.2 ± 0.00	1.0 ± 0.01
		24	1.0 ± 0.01	1.0 ± 0.00	1.0 ± 0.00	1.0 ± 0.00
2-Nonen-1-ol + linalool	12.68	6	26.0 ± 0.12	36.0 ± 0.29	26.0 ± 0.12	48.0 ± 0.08
		24	26.0 ± 0.11	78.0 ± 0.04	48.0 ± 0.46	68.0 ± 0.38
Ethyl benzoate	15.25	6	55.0 ± 0.44	95.0 ± 0.05	73.0 ± 0.22	87.0 ± 0.28
		24	45.0 ± 0.20	68.0 ± 0.41	28.0 ± 0.18	78.0 ± 0.64
Diethyl succinate	15.66	6	34.0 ± 0.38	69.0 ± 0.30	48.0 ± 0.03	89.0 ± 0.36
		24	68.0 ± 0.61	45.0 ± 0.25	65.0 ± 0.63	67.0 ± 0.27
Octanoic acid ethyl ester	17.02	6	7.0 ± 0.01	8.0 ± 0.07	9.0 ± 0.02	87.0 ± 0.79
		24	48.0 ± 0.30	68.0 ± 0.74	59.0 ± 0.18	71.0 ± 0.44
Ethyl acetal of benzaldehyde	18.13	6	23.0 ± 0.20	160.0 ± 1.18	120.0 ± 1.34	140.0 ± 0.19
		24	980.0 ± 5.99	680.0 ± 3.54	780.0 ± 3.09	470.0 ± 2.34
Diethyl malate	19.41	6	17.0 ± 0.08	580.0 ± 1.38	80.0 ± 0.33	180.0 ± 1.91
		24	150.0 ± 0.63	950.0 ± 3.87	870.0 ± 5.32	680.0 ± 2.69
Phenylacetalde-hyde diethyl acetal	22.24	6	6.0 ± 0.03	9.0 ± 0.08	9.0 ± 0.05	3.0 ± 0.03
		24	24.0 ± 0.04	48.0 ± 0.30	35.0 ± 0.07	78.0 ± 0.19
Methyl decanoate	22.55	6	20.0 ± 0.17	53.0 ± 0.40	35.0 ± 0.18	47.0 ± 0.04
		24	24.0 ± 0.03	48.0 ± 0.22	26.0 ± 0.10	87.0 ± 0.34
ar-Ionone	23.480	6	2.0 ± 0.01	78.0 ± 0.68	6.0 ± 0.03	59.0 ± 0.24
		24	20.0 ± 0.13	45.0 ± 0.26	65.0 ± 0.24	70.0 ± 0.73
Ethyl decanoate	24.593	6	4.0 ± 0.02	48.0 ± 0.29	3.0 ± 0.02	45.0 ± 0.50
		24	520.0 ± 4.71	68.0 ± 0.55	41.0 ± 0.20	78.0 ± 0.60
α-Ionone	24.954	6	5.0 ± 0.02	7.0 ± 0.07	9.0 ± 0.09	5.0 ± 0.02
		24	48.0 ± 0.51	48.0 ± 0.47	65.0 ± 0.05	36.0 ± 0.33
β-Ionone	25.749	6	9.0 ± 0.07	7.0 ± 0.00	7.0 ± 0.00	5.0 ± 0.04
		24	40.0 ± 0.27	50.0 ± 0.50	650.0 ± 6.03	98.0 ± 0.01
Total		6	9760.0 ± 59.6	12070.0 ± 12.3	10640.0 ± 78.3	9500.0 ± 55.9
		24	13030.0 ± 147.4	13860.0 ± 142.7	15430.0 ± 117.0	14650.0 ± 54.7

^1^ Values are expressed as the mean (*n* = 3) ± standard deviation.

**Table 7:** Changes in substances reacting with Folin-Ciocalteu reagent (FC) content and antioxidant activity ABTS, of sour cherry liqueurs during 24 weeks storage in temperatures 15 °C and 30 °C.

Test	Week	Temperature 15 °C		Temperature 30 °C	
		Without Sugar Added	With Sugar Added	Without Sugar Added	With Sugar Added
FC (mg GAE/mL)	0	1.16 ± 0.04 ^1^	1.16 ± 0.02	1.13 ± 0.01	1.15 ± 0.07
	6	1.15 ± 0.01	1.16 ± 0.06	1.12 ± 004	1.15 ± 0.01
	12	1.19 ± 0.04	1.18 ± 0.05	1.06 ± 0.06	1.18 ± 0.04
	18	1.21 ± 0.04	1.10 ± 0.03	0.98 ± 0.02	1.21 ± 0.03
	24	1.10 ± 0.03	1.21 ± 0.05	1.14 ± 0.01	1.25 ± 0.05
ABTS (µM TE/mL)	0	11.56 ± 0.29	11.77 ± 0.14	12.28 ± 0.64	12.62 ± 0.47
	6	11.56 ± 0.70	11.86 ± 0.23	11.46 ± 0.71	10.83 ± 0.61
	12	11.67 ± 0.28	11.30 ± 0.22	10.64 ± 0.38	8.66 ± 0.43
	18	11.51 ± 0.18	10.74 ± 0.39	9.47 ± 0.05	8.78 ± 0.07
	24	10.83 ± 0.29	10.68 ± 0.48	9.90 ± 0.32	7.85 ± 0.19

^1^ Values are expressed as the mean (*n* = 3) ± standard deviation.

**Table 8:** Analytical characteristics for determination of phenolic compounds.

Compound	Linear Range (µg/mL)	Equation of Calibration	Correlation Coefficient *R*²	LOD (µg/mL)	LOQ (µg/mL)
Cyanidin 3-*O*-glucoside	10–75	*y* = 0.8538*x*	0.9994	0.073	0.241
Quercetin 3-*O*-rutinoside	20–300	*y* = 1.9793*x*	0.9961	0.107	0.354
(+) Catechin	20–200	*y* = 4.1786*x*	0.9996	0.438	1.446
Chlorogenic acid	20–300	*y* = 0.9195*x*	0.9998	0.062	0.199

*y*—concentration, *x*—area.

## Supplementary Materials

The supplementary materials are available online. Figure S1: LC MS/MS chromatogram of of phenolic compounds in sour cherry liqueurs in positive (A) and negative mode (B), Table S1: LC-MS identification of phenolic compounds in sour cherry liqueurs, Table S2: Chromatographic data for phenolic compounds of cherry liqueurs, Table S3: Validation—intra-day and inter-day precision.
