# Supplementary material for: Phenolic Composition Stability and Antioxidant Activity of Sour Cherry Liqueurs
Source: Molecules. 2018 Aug 27;23(9):2156. doi: 10.3390/molecules23092156 (PMC6225465; doi:10.3390/molecules23092156)

Figure S 1. LC MS/MS chromatogram of of phenolic compounds in sour cherry liqueurs in positive (A) and negative mode (B)

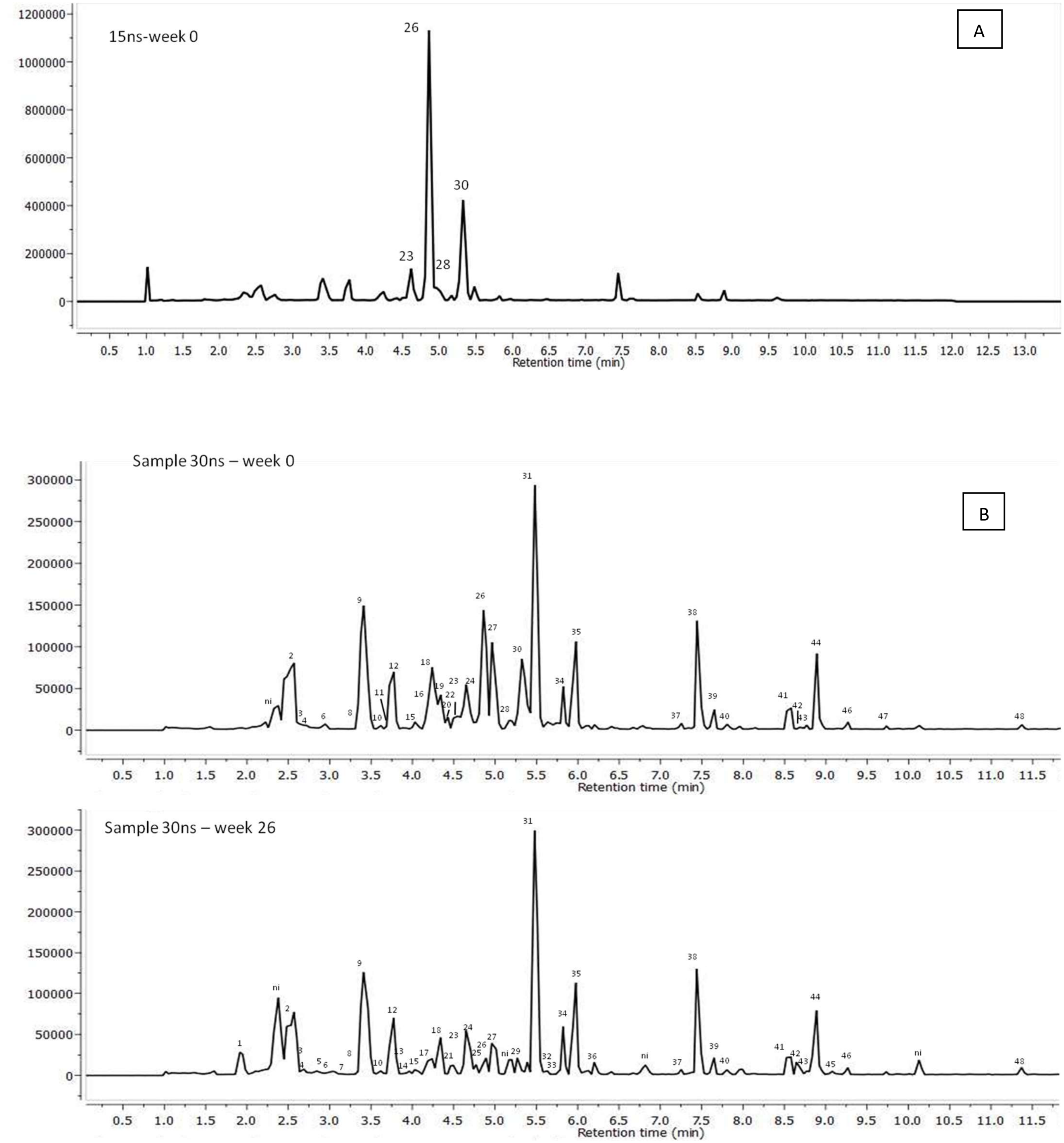

Supplement: Supplementary file 1 [file molecules-23-02156-s001.zip › supplementary-proofed-pdf/Supplement Figure S1.pdf]
