# Supplementary material for: Phenolic Composition Stability and Antioxidant Activity of Sour Cherry Liqueurs
Source: Molecules. 2018 Aug 27;23(9):2156. doi: 10.3390/molecules23092156 (PMC6225465; doi:10.3390/molecules23092156)
Supplement: Supplementary file 1 [file molecules-23-02156-s001.zip › supplementary-proofed-pdf/Supplement Table S1.pdf]

Table S 1. LC-MS identification of phenolic compounds in sour cherry liqueurs

			MS	MS/MS	Week					
Peak no	Tentative assingment	t_R (min)	$[M-H]^-$/ $[M-H]^+$ [*m/z*]	fragment jons [*m/z*]	0	24				Reference
						15ns	30ns	15s	30s	
1	Protocatechuic acid hexoside	1.91	315.0717	153.0195/ 109.0292	x	x	x	n	n	(Seeram, Bourquin, & Nair, 2001)
2	Neochlorogenic acid	2.45	353.0879	191.0553/179.0349/135.0443	x	x	x	x	x	(Bonerz, Würth, Dietrich, & Will, 2007; Cao, Jiang, Lin, Li, Sun, & Chen, 2015)
3	Dicaffeoylquinic acid 1	2.57	515.1381	353.0879/191.0553/179.0349/135.0443/	tr	x	x	tr	n	Putatively characterized
4	Dicaffeoylquinic acid 2	2.69	515.14	179.0343/191.0562	x	tr	tr	n	n	Putatively characterized
5	Caffeic acid hexoside 1	2.71	341.0887	179.0349	x	tr	x	n	n	Putatively characterized
6	Kaempferol -3- hexoside	2.91	771.204	609.1342/	x	tr	x	x	x	Putatively characterized
7	Flavan-3-ol dimer (procyanidin B2)	2.94	577.1349	289.0723	x	x	x	x	x	(Bonerz, Würth, Dietrich, & Will, 2007)
8	Flavan-3-ol monomer ((+)catechin)	3.25	289.0709						x	(Bonerz, Würth, Dietrich, & Will, 2007; Cao, Jiang, Lin, Li, Sun, & Chen, 2015)
9	p-Coumaroylquinic acid 1	3.41	337.0899	675.1927/163.0406/119.0510	x	x	x	x	x	(Cao, Jiang, Lin, Li, Sun, & Chen, 2015; Picariello, De Vito, Ferranti, Paolucci, & Volpe, 2016)
10	Caffeic acid hexoside 2	3.62	341.0887	179.0349/135.0440	x	x	x	x	x	Putatively characterized
11	Flavan-3-ol dimer	3.65	577.1349	289.0723	x	x	n	x	n	Putatively characterized
12	Chlorogenic acid	3.77	353.0879	707.1852/191.0553	x	x	x	x	x	(Bonerz, Würth, Dietrich, & Will, 2007; Cao, Jiang, Lin, Li, Sun, & Chen, 2015)
13	Flavan-3-ol trimer	3.95	865.1841	577.1272/289.0352	x	x		x	x	Putatively characterized
14	Flavan-3-ol monomer((-) epicatechin)	3.98	289.0352				x			(Nowicka & Wojdyło, 2016)
15	benzoic acid dihexoside	4.03	445.1333	323.0978/121.0288	x	x	x	x	x	(Seeram, Bourquin, & Nair, 2001; Toydemir, Capanoglu, Gomez Roldan, de Vos, Boyacioglu, Hall, et al., 2013)
16	Flavan-3-ol trimer	4.07	865.1984	577.1349/289.0723/125.0241	x	x	x	n	n	Putatively characterized
17	Flavan-3-ol dimer	4.18	577.1351	407.0778/ 289.0735	tr	x	x	x	x	Putatively characterized
18	Kaempferol derivative	4.27	773.2153	285.0394/125.0241/637.2014/163.0400	x	x	x	x	x	Putatively characterized
19	apigenin or genistein hexoside	4.34	431.1547	269.1006/161.0449	x	x	x	x	x	Putatively characterized
20	Kaempferol dihexsoside derivative	4.43	627.1564	491.1386/285.0394/	x	x	x	n	n	Putatively characterized
21	Vanilin	4.5	153.0189	109.029	n	x	x	n	n	Putatively characterized
22	Kaempferol dihexoside	4.55	611.1603	465.1037/491.1386/323.1346/285.0394	x	x	x	x	n	Putatively characterized
23	Cyanidin 3-sophoroside	4.6	609.1432 (+) 611.1612	285.0394 (+) 287.0554	x	x	x	x	n	(Cao, Jiang, Lin, Li, Sun, & Chen, 2015)
24	Amygdalin	4.65	456.1516	502.1583/323.0978/369.1028	x	x	x	x	x	(Toydemir, et al., 2013)
25	p-Coumaroylquinic acid 2	4.77	337.0924	163.0406/119.0510	tr	x	x	x	x	(Bonerz, Würth, Dietrich, & Will, 2007; Picariello, De Vito, Ferranti, Paolucci, & Volpe, 2016)

26	Cyanidin 3-(2^G glucosyl rutinoside)	4.86	755.2062 (+) 757.2199	285.0394/284.0313 (+) 287.0551	x	x	x	x	x	(Cao, Jiang, Lin, Li, Sun, & Chen, 2015)
27	Flavan-3-ol monomer	4.96	289.0723	289.0723	x	x	x	x	x	Putatively characterized
28	Cyanidin 3-glucoside	5.05	447.0916 (+) 449,1083	285.0394/284.0313 (+) 287.0554	x	x	tr	x	tr	(Cao, Jiang, Lin, Li, Sun, & Chen, 2015)
29	Coumaroylquinic acid	5.27	337.0924	163.0406/119.0510		n	x	n	x	Putatively characterized
30	Cyanidin 3-rutinoside	5.34	593.1507 (+) 595.1667	285.0395 (+)287.0555	x	x	tr	x	x	(Cao, Jiang, Lin, Li, Sun, & Chen, 2015)
31	genistein or **apigenin** (269)-pentoside	5.48	447.1552	401.144 /803.3015/ 269.1039/161.0449	x	x	x	x	x	Putatively characterized
32	Flavan-3-ol trimer	5.63	863.1810	577.1349/289.0723	x	x	n	n	n	Putatively characterized
33	Flavan-3-ol trimer	5.72	577.134	1153.261/289.0723	x	x	n	n	n	Putatively characterized
34	Kaempferol trihexoside	5.87	771.2018	285.0394	x	x	x	x	x	Putatively characterized
35	Genistein or **apigenin**-rhamnoside	5.98	461.166	415.1604/269.1039/161.0449/101.0232	x	x	x	x	x	Putatively characterized
36	Quercetin 3-(2G-glucosylrutinoside)	6.14	771.2018	625.1386/609.1432/463.0287/301.0354	x	x	x	x	n	(Bonerz, Würth, Dietrich, & Will, 2007)
37	Naringenin - hexoside (prunin)	7.25	433.1118	271.0609/151.0070	x	x	x	x	n	Putatively characterized
38	Quercetin - rutinoside	7.46	609.1483	301.0354	x	x	x	x	x	(Bonerz, Würth, Dietrich, & Will, 2007; Cao, Jiang, Lin, Li, Sun, & Chen, 2015)
39	Quercetin - glucoside	7.61	463.0887	301.0319	x	x	x	x	x	(Bonerz, Würth, Dietrich, & Will, 2007; Cao, Jiang, Lin, Li, Sun, & Chen, 2015)
40	Genistein or apigenin derivative	7.8	441.1738	269.0435	x	x	tr	x	n	Putatively characterized
41	Kaempferol rutinoside	8.58	593.1507	285.0394	x	x	x	x	x	(Bonerz, Würth, Dietrich, & Will, 2007; Cao, Jiang, Lin, Li, Sun, & Chen, 2015)
42	Isorhamnetin rutinoside (or galactoside-rhamnoside)	8.67	623.1598	315.0512	x	x	x	x	x	(Toydemir, et al., 2013)
43	Kaempferol glucoside	8.77	447.0916	285.0394/284.0313	x	x	x	x	x	(Cao, Jiang, Lin, Li, Sun, & Chen, 2015)
44	Isorhamnetin rutinoside	8.89	623.1598	315.0504/271.0597	x	x	x	x	x	(Bonerz, Würth, Dietrich, & Will, 2007; Cao, Jiang, Lin, Li, Sun, & Chen, 2015)
45	Isorhamnetin glucoside	9.15	477.1022	315.0504/	x	x	x	n	n	(Picariello, De Vito, Ferranti, Paolucci, & Volpe, 2016)
46	Eriodictyol or dehydrokaempferol derivative	9.22	503.1766	287.0558/151.0034	x	x	x	x	x	Putatively characterized
47	Caffeoyl-p-coumaroylquinic acid	10.37	499.1242	337.0920/ 191.0553/163.0411	x	n	n	n	n	Putatively characterized
48	Quercetin	11.37	301.0354	151.0017	x	x	x	x	x	(Toydemir, et al., 2013)

x-present
tr-traces
n-not detected
ni - not identified

Putatively characterized - compound classes assigned by comparison of the elemental composition to public and commercial databases and/or based on fragmentation, UV absorption and retention time corresponding to compounds described in the literature

Bonerz, D., Würth, K., Dietrich, H., & Will, F. (2007). Analytical characterization and the impact of ageing on anthocyanin composition and degradation in juices from five sour cherry cultivars. *European Food Research and Technology, 224*(3), 355-364.

Cao, J., Jiang, Q., Lin, J., Li, X., Sun, C., & Chen, K. (2015). Physicochemical characterisation of four cherry species (Prunus spp.) grown in China. *Food Chemistry, 173*, 855-863.

Nowicka, P., & Wojdyło, A. (2016). Stability of phenolic compounds, antioxidant activity and colour through natural sweeteners addition during storage of sour cherry puree. *Food Chemistry, 196*, 925-934.

Picariello, G., De Vito, V., Ferranti, P., Paolucci, M., & Volpe, M. G. (2016). Species- and cultivar-dependent traits of Prunus avium and Prunus cerasus polyphenols. *Journal of Food Composition and Analysis, 45*, 50-57.

Seeram, N. P., Bourquin, L. D., & Nair, M. G. (2001). Degradation Products of Cyanidin Glycosides from Tart Cherries and Their Bioactivities. *Journal of Agricultural and Food Chemistry, 49*(10), 4924-4929.

Toydemir, G., Capanoglu, E., Gomez Roldan, M. V., de Vos, R. C. H., Boyacioglu, D., Hall, R. D., & Beekwilder, J. (2013). Industrial processing effects on phenolic compounds in sour cherry (Prunus cerasus L.) fruit. *Food Research International, 53*(1), 218-225.
