# Supplementary material for: Phenolic Composition Stability and Antioxidant Activity of Sour Cherry Liqueurs
Source: Molecules. 2018 Aug 27;23(9):2156. doi: 10.3390/molecules23092156 (PMC6225465; doi:10.3390/molecules23092156)
Supplement: Supplementary file 1 [file molecules-23-02156-s001.zip › supplementary-proofed-pdf/Supplement table S2.pdf]

Table S21. Chromatographic data for phenolic compounds of cherry liqueurs

Peak Nº	Compound	RT [min]	λ max [nm]	Spectrum
	**Phenolic acids**			
1	Neochlorogenic acid	2.78	245. 325	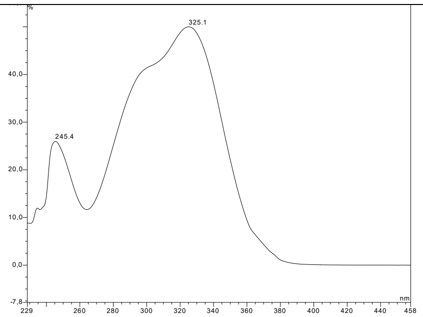
2	Coumaroilquinic acid	2.88	311	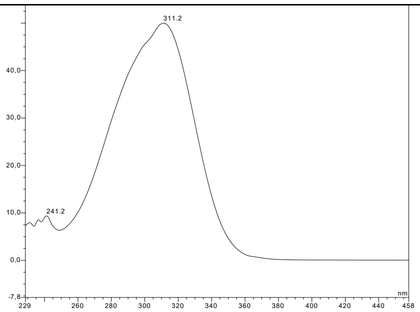
3	Chlorogenic acid	5.27	245.326	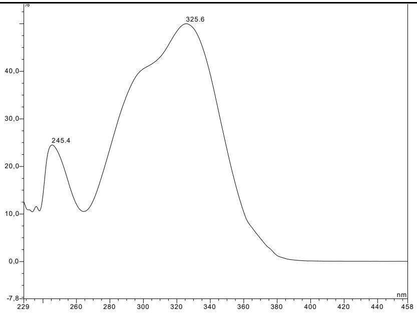
	**Anthocyanins**			
4	Cyanidin 3 O- sophoroside	5.89	281.516	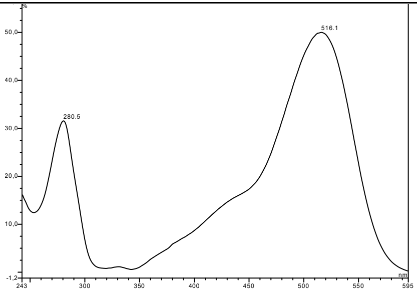
5	Cyanidin 3- O- glucosyl rutinoside	6.38	281.517	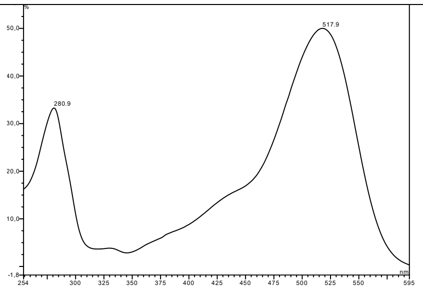

6	Cyanidin 3- O- glucoside	6.82	281. 515	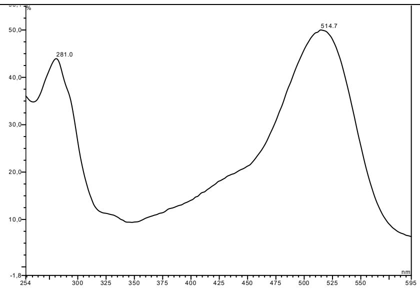
7	Cyanidin 3- O- sambubioside-5-rhamnoside	7.08	280.0519	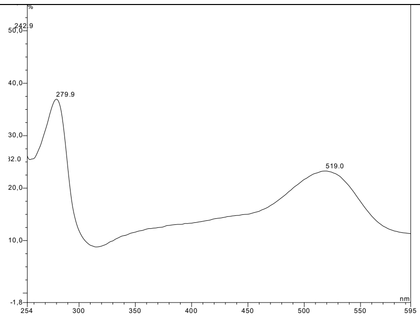
8	Cyanidin 3- O- rutinoside	7.47		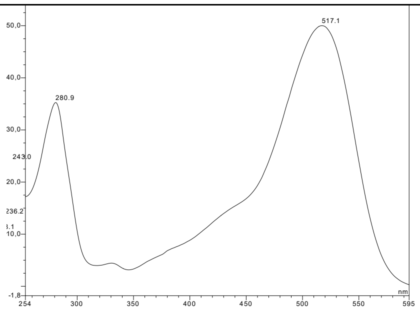
	**Flavan 3-ols**			
9	Procyanidin B1	3.17	243.278	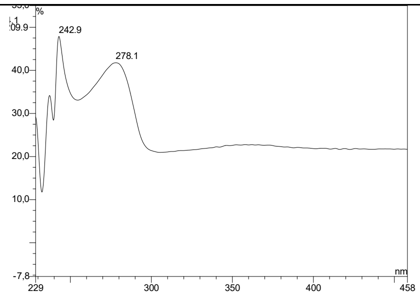
10	Procyanidin B2	5.43	243.282	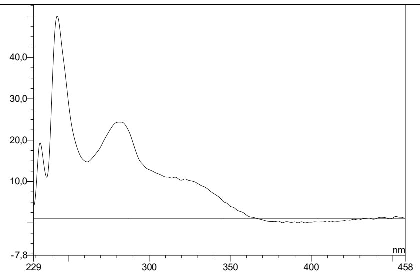

11	(-)Epicatechin +dimer	7.46	243.281	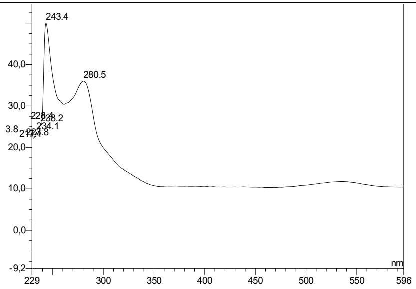
12	Procyanidin C1+tetramer	8.37	243.280	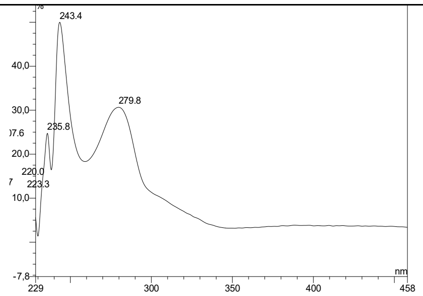
	**Flavonols**			
13	Kaempferol- trihexoside 1	5.51	339	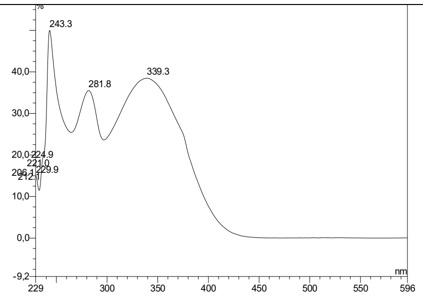
14	Kaempferol- trihexoside 2	5.94	255.342	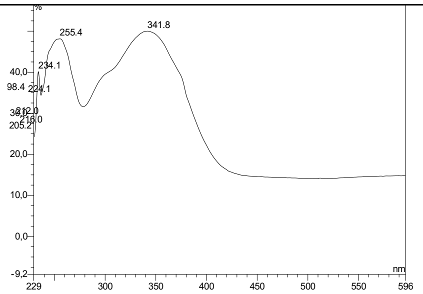
15	Kaempferol- dihexoside	8.76	253. 344	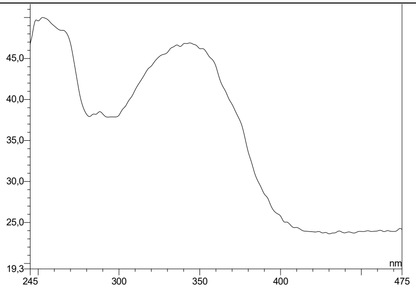

16	Quercetin-rutinoside-rhamnoside	9.40	256. 354	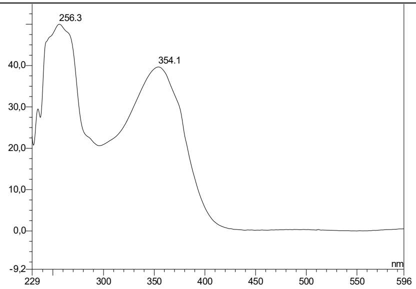
17	Quercetin-rutinoside	12.00	258. 350	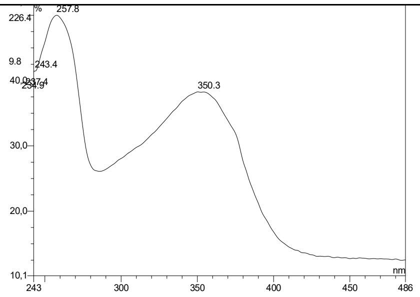
18	Quercetin -glucoside	12.12	257. 350	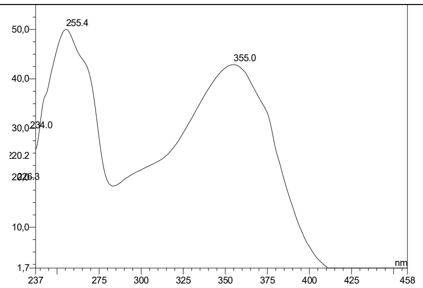
19	Kaempferol - rutinoside	14.17	266. 346	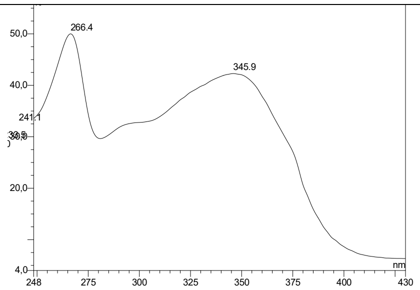
20	Isorhamnetine- rutinoside	14.8	255. 355	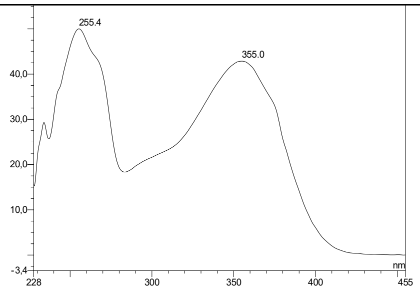
21	Quercetin	20.20	257.373	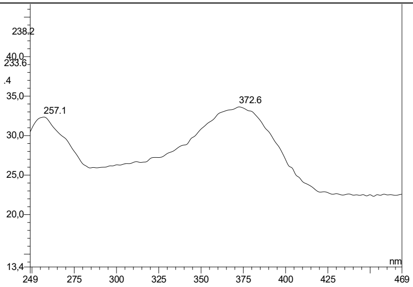

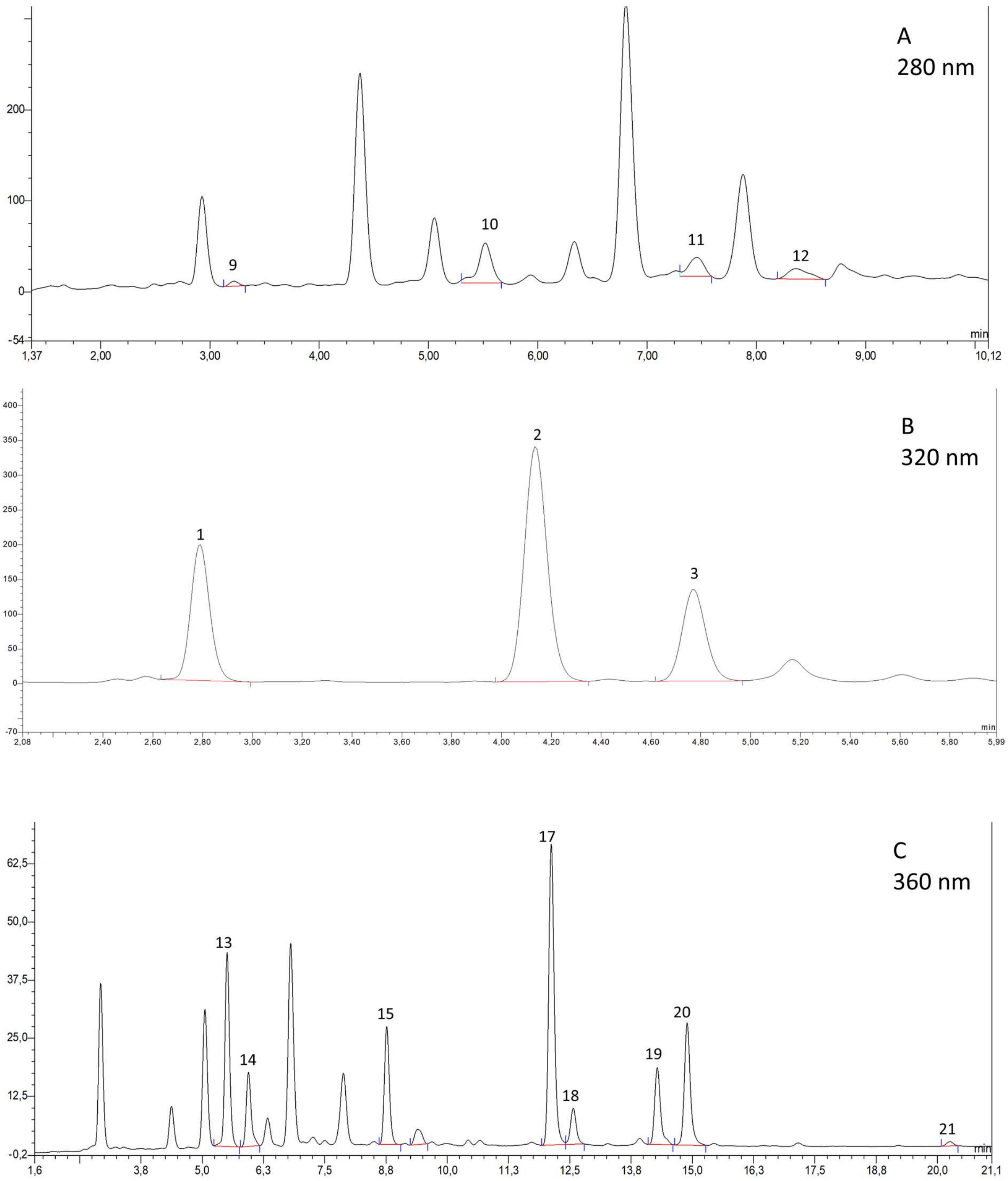
A
280 nm
9
10
11
12
min
B
320 nm
1
2
3
min
C
360 nm
13
14
15
17
18
19
20
21
min

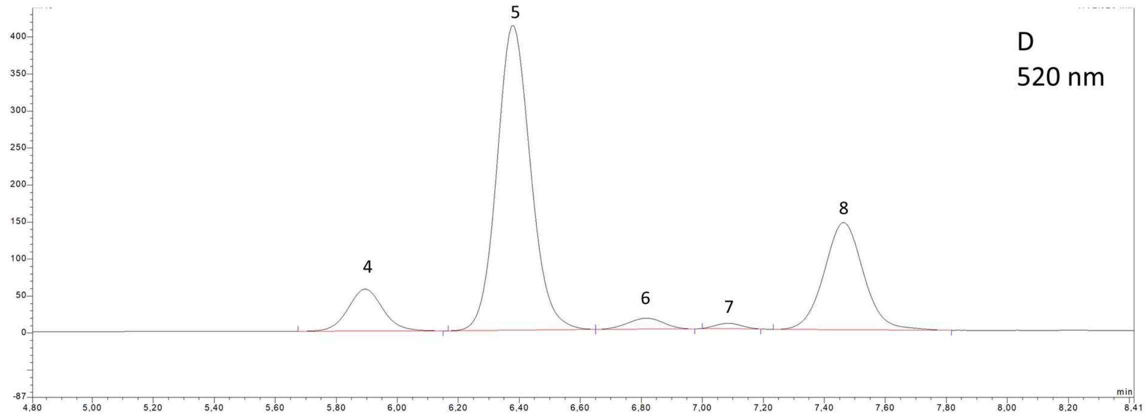

Figure 1. HPLC chromatograms of phenolic compounds (A- flavan 3-ols; B-phenolic acids; C-flavonols and D-anthocyanins)
