# Supplementary material for: Phenolic Composition Stability and Antioxidant Activity of Sour Cherry Liqueurs
Source: Molecules. 2018 Aug 27;23(9):2156. doi: 10.3390/molecules23092156 (PMC6225465; doi:10.3390/molecules23092156)
Supplement: Supplementary file 1 [file molecules-23-02156-s001.zip › supplementary-proofed-pdf/Supplement table S3.pdf]

Table S3. Validation – intra-day and inter-day precision

Compound		Concentration µg/ml	Intraday mean±SD µg/ml	Intraday RSD (%)	Intraday Accuracy (%)	Interday mean±SD µg/ml	Interday RSD (%)	Interday Accuracy (%)
Chlorogenic acid	day 1	20	21.79±0.52	3.41	108.94	21.19±0.62	2.94	105.94
	day 2	20	20.54±0.05	0.36	102.72			
	day 3	20	21.23±0.37	2.53	106.15			
	day 1	100	98.02±0.95	1.38	98.02	99.42±1.29	1.29	99.42
	day 2	100	99.68±0.88	1.27	99.68			
	day 3	100	100.56±1.90	2.70	100.56			
	day 1	300	306.69±1.95	0.91	102.23	302.45±	1.30	99.19
	day 2	300	301.76±1.78	0.85	100.59			
	day 3	300	298.90±1.80	0.86	99.63			
Quercetin 3-rutinoside	day 1	20	19.89±0.25	2.53	99.46	19.72±0.15	0.76	98.60
	day 2	20	19.61±0.18	1.86	98.07			
	day 3	20	19.65±0.06	0.57	98.27			
	day 1	100	100.27±0.76	1.51	100.27	100.47±0.73	0.73	100.47
	day 2	100	101.28±1.17	2.29	101.28			
	day 3	100	99.86±0.78	1.54	99.86			
	day 1	300	299.86±2.33	2.33	99.95	298.61±1.09	0.37	99.54
	day 2	300	298.08±0.99	0.66	99.36			
	day 3	300	297.88±0.71	0.47	99.29			
Cyanidin 3-glucoside	day 1	10	9.6±0.35	3.14	96.05	9.70±0.24	2.45	103.12
	day 2	10	9.52±0.49	4.44	95.20			
	day 3	10	9.97±0.45	3.82	99.68			
	day 1	50	48.15±2.26	4.01	96.31	49.05±0.80	1.63	98.10
	day 2	50	49.31±1.77	3.06	98.61			
	day 3	50	49.69±2.40	4.13	99.38			
	day 1	75	75.56±2.12	2.40	100.75	74.37±1.09	1.47	99.15
	day 2	75	73.4±2.83	3.29	97.90			
	day 3	75	74.11±1.70	1.96	98.81			
(+) Catechin	day 1	20	20.68±0.35	7.14	103.42	20.29±0.38	1.86	98.55
	day 2	20	19.932±1.16	3.26	99.66			
	day 3	20	20.27±0.11	2.33	101.33			
	day 1	100	101.96±0.99	4.06	101.96	100.50±2.01	2.00	100.50
	day 2	100	101.33±1.06	4.37	101.33			
	day 3	100	98.20±0.85	3.61	98.20			
	day 1	200	204.54±1.34	2.74	102.27	201.205±2.96	1.47	100.60
	day 2	200	198.90±1.70	3.57	99.45			
	day 3	200	200.15±1.41	2.95	100.08			

RSD-relative standard dewiation
